# Implementing a structured education program for children with diabetes: lessons learnt from an integrated process evaluation

**DOI:** 10.1136/bmjdrc-2014-000065

**Published:** 2015-04-28

**Authors:** Mary Sawtell, Liz Jamieson, Meg Wiggins, Felicity Smith, Anne Ingold, Katrina Hargreaves, Meena Khatwa, Lucy Brooks, Rebecca Thompson, Deborah Christie

**Affiliations:** 1 Social Science Research Unit, UCL Institute of Education, London, UK; 2 Department of Practice and Policy, UCL School of Pharmacy, London, UK; 3 Social Science Research Unit, Institute of Education, London, UK; 4 Medical Statistics Department, London School of Hygiene and Tropical Medicine, London, UK; 5 University College London Hospitals NHS Foundation Trust, London, UK

**Keywords:** Pediatric Diabetes, Education, Randomized Controlled Trial, Research Methodologies

## Abstract

**Background:**

There is recognition of an urgent need for clinic-based interventions for young people with type 1 diabetes mellitus that improve glycemic control and quality of life. The Child and Adolescent Structured Competencies Approach to Diabetes Education (CASCADE) is a structured educational group program, using psychological techniques, delivered primarily by diabetes nurses. Composed of four modules, it is designed for children with poor diabetic control and their parents. A mixed methods process evaluation, embedded within a cluster randomized control trial, aimed to assess the feasibility, acceptability, fidelity, and perceived impact of CASCADE.

**Methods:**

28 pediatric diabetes clinics across England participated and 362 children aged 8–16 years, with type 1 diabetes and a mean glycosylated hemoglobin (HbA1c) of 8.5 or above, took part. The process evaluation used a wide range of research methods.

**Results:**

Of the 180 families in the intervention group, only 55 (30%) received the full program with 53% attending at least one module. Only 68% of possible groups were run. Staff found organizing the groups burdensome in terms of arranging suitable dates/times and satisfactory group composition. Some staff also reported difficulties in mastering the psychological techniques. Uptake, by families, was influenced by the number of groups run and by school, work and other commitments. Attendees described improved: family relationships; knowledge and understanding; confidence; motivation to manage the disease. The results of the trial showed that the intervention did not significantly improve HbA1c at 12 or 24 months.

**Conclusions:**

Clinic-based structured group education delivered by staff using psychological techniques had perceived benefits for parents and young people. Staff and families considered it a valuable intervention, yet uptake was poor and the burden on staff was high. Recommendations are made to inform issues related to organization, design, and delivery in order to potentially enhance the impact of CASCADE and future programs.

**Current Controlled Trials:**

ISRCTN52537669.

**Key messages**

- The Child and Adolescent Structured Competencies Approach to Diabetes Education (CASCADE) structured education program is perceived by young people and parents who attend as having benefits but practical challenges associated with attendance result in low uptake.
- Staff are positive about the potential of the program but organizational aspects are unacceptably burdensome.
- CASCADE is potentially deliverable to families as part of routine care and could be a useful intervention. However, improvements in clinical and administrative support, staff training, program content, and service structures are required to ensure fidelity to the program and feasibility and acceptability to key stakeholders.

## Introduction

Type 1 diabetes mellitus (T1DM) in children and young people is increasing worldwide. Fewer than one in six children and young people achieve glycosylated fraction of hemoglobin (HbA1c) values in the range identified as providing best future outcomes.^1^ It has been recognized that there is an urgent need for clinic-based pragmatic, feasible, and effective interventions that improve both glycemic control and quality of life, with a particular emphasis on structured education programs.^2^ In recent years, a number of large multicenter studies have trialed a standard education intervention.^3–5^ Findings published, to date, report no significant positive impact on glycemic control as measured by HbA1c and only limited impact on a wide range of secondary measures.^4^ ^5^ Nevertheless, the recent Best Practice Tariff for Paediatric Diabetes for diabetes services in the UK^6^ requires the provision of structured educational programs for young people and their families and, as a consequence, there is an urgent need for high-quality evidence to inform the implementation of this recommendation.

The CASCADE (Child and Adolescent Structured Competencies Approach to Diabetes Education) pragmatic cluster randomized controlled trial (RCT) with integral process and economic evaluation is the most recent study. It was undertaken by a team that included clinicians from a London-based pediatric diabetes clinic, a representative from a diabetes patient organization and researcher teams from three universities in London. The CASCADE intervention is a structured education program designed for children and young people with T1DM aged between 8 and 16 years and their parents or carers.^7^ The intervention underwent phase 1 pilot work and a non-randomized trial, in which the delivery was carried out by a psychologist.^8^ The CASCADE intervention was then modified to be delivered by two members of a diabetes multidisciplinary team (MDT) who receive 2 days of training to enable them to become ‘site educators’. CASCADE is a manual-based program. It is delivered in four modules over 4 months, each lasting approximately 2 hours, to groups of three to four families with children and young people grouped according to age (8–11 or 12–16 years). Two psychological approaches, motivational interviewing and solution-focused brief therapy, shown to have potential with children with diabetes are central to the CASCADE intervention.^9^ ^10^ These aim to engage participants to identify and develop their own positive approaches and consequent behavior change relevant to the management of their condition. The intervention thus offers both structured education, to ensure young people (and their parents) know what they need to know, and a delivery model designed to motivate self-management through empowerment techniques (see table 1).

**Table 1:** Outline of the CASCADE program (as set out in the manual)

**The teaching plan**	Session activities, objectives, time guides, and resources including key information essential for the educator, learning objective for the family, and brief descriptions of each activity

Each module starts with a review of, and since, the previous session, creating an opportunity for families to highlight any changes that have taken place and to congratulate young people on successes	
**Module 1**	Focuses on the relationship between food, insulin, and BG (*eg, considering the pros and cons of matching insulin to food to attain better glycemic control*)
**Module 2**	Reviews BG testing and factors influencing BG fluctuation (*eg, identifying factors that cause BG to rise and fall and explore hypoglycemia definitions, reviewing symptoms according to severity)*
**Module 3**	Looks at the pros and cons of adjusting insulin *(eg, a brainstorming session considers when, how, and who to contact for help managing hyperglycemia)*
**Module 4**	Addresses aspects of living with diabetes, including managing BG levels and exercise (*eg, young people and families complete a ‘blueprint for success’. This marks the end of the sessions and acknowledges the steps into the future the young person has already made)*
Homework tasks are given to families to consolidate learning after each module	

BG, blood glucose; CASCADE, Child and Adolescent Structured Competencies Approach to Diabetes Education.

The intention is that delivering CASCADE to groups will provide staff with an alternative mode of working with young people in the clinic setting to improve outcomes, rather than requiring additional work.

## Cascade trial summary

The trial involved young people with T1DM and family members in 28 English pediatric diabetes clinics (randomly assigned at clinic level to intervention or control) in London, South East England, and the Midlands. Clinics eligible to participate were staffed by at least one pediatrician and pediatric nurse with an interest in diabetes. Other inclusion criteria included not running a group education program at time of recruitment and not participating in a similar pediatric diabetes trial within the past 12 months. It was approved by the University College London (UCL)/UCLH Research Ethics Committee (REC) reference number 07/HO714/112. Site-specific approval was granted at each site. Three hundred and sixty-two young people were recruited to the study. Inclusion criteria included: diagnosis with a duration ≥12 months; mean 12-month HbA1c of 8.5 or above; aged 8–16 years. Clinical staff identified eligible young people from their patient list. Researchers sent letters and information sheets to these young people and their parents or carers inviting them to participate in the research and to speak to a researcher at their next clinical appointment. Recruitment was primarily carried out by members of the process evaluation team who attended clinics at which eligible young people had an appointment. Signed consent forms were collected from parents and children wishing to participate.

The primary outcome measure was venous HbA1c at 12 and 24 months. Secondary outcomes included: knowledge, skills and responsibilities associated with diabetes management; emotional and behavioral adjustment; quality of life. Two staff members from each intervention site clinical team participated in the 2 days CASCADE training program. These site educators then took responsibility for organizing the modules at their clinics and delivering the intervention.

The extensive and integral process evaluation was designed to enable an understanding of the implementation of CASCADE and examination of the interaction of causal mechanisms and contextual factors that may be determinants of the intervention's success or failure, as assessed by the trial.^11^ Given that the trial found no evidence of benefits on venous HbA1c at 12 and 24 months and little evidence of benefits on secondary outcomes, the focus of this paper is to use the findings of the process evaluation to suggest how future structured education may be more effectively implemented.^12^

## Process evaluation methods

The process evaluation aimed to assess the feasibility, acceptability, fidelity and perceived impact of the CASCADE intervention. It ran for the 4-year life of the trial and included the multiple methods shown in table 2. Researchers from the process evaluation teams at the Institute of Education (IOE) and the School of Pharmacy (SOP) conducted the fieldwork.

**Table 2:** Process evaluation methods and response rates

Phase of the study	Methods	Purpose of methods	Response rates
Two-day training of site educators	Unstructured observation of training of site educators by a member of the research team	Fidelity of training	6 training days observed
	Participant questionnaires (completed 2 weeks after training)	Description of participants Participant experience/acceptability of training	27 participant questionnaires from 18 nurses, 8 dietitians, and 1 doctor (63% of participants)
	Semistructured interviews with the two trainers	Background to intervention development; views on training days	Both trainers
Delivery of CASCADE modules with patients/carers	Observation of modules carried out by a member of research team including rating of fidelity to psychological techniques and content of manual	Fidelity of delivery Experience/acceptability of delivery of program to site educators Experience/acceptability of participation in the program by young people/parents	47 CASCADE modules observed across 13 intervention sites (12 each of modules 1, 2, and 4; 11 of module 3)
	Self-complete feedback proformas for site educators	Who delivered each module; who attended each module Self-assessment of delivery fidelity and general feedback on each module	Site educators returned 125 feedback proformas (94% of 131 completed modules)
Following delivery of all CASCADE groups	Young person and parent 12 and 24 month questionnaires in intervention arm	Perceptions of impact Acceptability of the intervention	Process questions were completed on questionnaires by 135 young people (82%) and 121 parents (66%) at 12 months; 121 young people (66%) and 114 (63%) parents at 24 months
	Semistructured interviews (audio-recorded) with site staff (nurses and dietitians), young people, and parents/carers in both trial arms	Description of standard care—including any structured education currently delivered Intervention arm only—experiences of the intervention (training and delivery)	30 site staff (16 intervention sites; 14 control) 53 young people (32 intervention/21 control) and 52 parents were interviewed. Of the young people, 31 were female; 17 were 10–11 years old; and 36 were 12–18 years old

CASCADE, Child and Adolescent Structured Competencies Approach to Diabetes Education.

## Process evaluation data analysis

Qualitative data analysis was carried out by the process evaluation teams at IOE and SOP (all the authors except LB, RT, and DC). Qualitative analysis of the interview data, supported by the use of NVivo software, identified key topics and issues that emerged through familiarization with transcripts.^13^ Pertinent excerpts were coded and memos written to summarize and synthesize emerging themes. Researchers refined their analysis ensuring that themes were crosschecked with other data, first within and then between transcripts. Analysis of each training workshop observation was carried out by a researcher, who was not the observer, reading through the notes made by the observer and identifying key themes and fidelity issues emerging from the data. Quantitative data were analyzed by MW using Excel and the SPSS V.19 software for statistical tests. In terms of the CASCADE modules delivered in the sites, composite fidelity delivery scores were created for content and for technique from individual researcher observer and site educator self-rated scores. A further composite variable was then calculated which summed the content and technique scores for each site across all four modules, allowing comparison across sites and modules.

## Process evaluation results

The results are structured under the following themes: recruitment and training of site educators; organizing the groups; delivery of the modules; uptake and acceptability of the modules; and perceptions of impact. Response rates are reported in table 2.

### Recruitment and training of site educators

The National Institute for Health and Care Excellence (NICE) requirement,^2^ that structured education programs are delivered as part of routine care was widely recognized by clinic staff and, as a consequence, it proved relatively straightforward to recruit two members of the MDT from each of the 14 intervention sites to become site educators. The majority of site educators were experienced pediatric diabetes specialist nurses (PDSNs); in approximately half of the sites one of the educators was a dietitian. The diabetes specialist nurse and psychologist who developed the intervention delivered the 2 day CASCADE training for site educators in four workshop sessions. In general, it was feasible for sites to send the required minimum of two staff to the core workshops. A few sites sent additional interested members of the MDT though only four consultants attended some or all of the training. The training was delivered in a central London location, except for one site where following a request, training was delivered locally. Site staff reported this change in location to be helpful. The majority of staff who completed the questionnaire following the workshops indicated they had been ‘extremely’ or ‘very’ keen to participate. Most staff thought the training was very good, motivating, and comprehensive.

The most common concern raised in staff interviews about becoming site educators and running the CASCADE program, both before and after the training, was additional workload. Other concerns included practical constraints such as finding available rooms in which to run the groups and ability to rapidly change their practice to employ the psychological approaches underpinning CASCADE. One site educator commented.

It [the training] was a lot in the few days. Teaching people theories and expecting them to suddenly change their behaviour I think is very difficult.

The two trainers, and some attendees, expressed concern about levels of diabetes knowledge among the site educators.

Some of it [the training] was ending up teaching them the content as opposed to teaching them the style of delivery. (UCLH trainer)

At the time [of the training] I'd got very little diabetes knowledge so, for me, I was actually learning from it and I know that's not really what it was about but a lot of it was that…I found it quite intimidating because of my lack of knowledge. (Site educator)

### Organizing the groups

A total of 30 complete CASCADE groups, comprising all four modules, were run across 12 of the 14 intervention sites. A post hoc calculation, based on the number of study recruits in a site and the optimum group size of 3–4 young people, suggested 44 groups should have been run across the 14 intervention sites. Thus, 68% of possible groups ran, with only three clinics completing the maximum number of groups possible for their site. A key reason for this limited delivery was difficulties with organizing the groups. The organization was undertaken by the site educators in all the sites. This involved: deciding which participants should be grouped together using similar ages as a key criterion; setting dates and times; inviting families to attend; and booking a room. Interviews revealed that site educators found these processes frustrating and very time-consuming. One site educator commented:

I didn't notice that it saved me any time because I was constantly chasing them [families] up to be there.

One site delivered no modules because the lead site educator left her PDSN post soon after the training. Another site delivered only the first module because of a number of challenges which included: the small number of potential eligible patients on the clinic list; poor uptake of the first module by young people/parents; practical organizational constraints.

All the sites ran the groups in addition to routine clinics where standard care continued to be received by patients on an individual basis. Staff interview data revealed that the pressure on hospital clinic facilities was too great to make running the groups feasible during clinics. Establishing a date and time for the group sessions that was acceptable to the families was extremely challenging. To maximize attendance, some site educators tried a range of timings including during school hours, after school, weekends, and school holidays. Communication with families, about groups, was via a combination of letter, telephone, and (occasionally) text messages. No sites used email or online meeting booking sites. Despite all the negotiation and careful planning by site educators, late cancellation or non-attendance by participants was reported as common.

Some didn't even bother to get back to us and some did and said they were still gonna come but still didn't come. It is frustrating and I think that's what was time consuming, which I hadn't really accounted for…(Site educator)

As a result of these difficulties, compromises were made to the intended group size and composition. Groups often had small numbers (sometimes one family only) and/or a wide age range among the young people attending. Although the intention was to run four modules with the same participants, the composition of many groups changed.

### Delivery of the modules

The site educators believed they were appropriate individuals to deliver the intervention because they knew patients well, although familiarity with patients was not a requirement. Participating families appeared to support this view. All sites had continuity of at least one trained site educator, but complications in sustaining the availability of a second educator in a few sites resulted in some lack of continuity of trainer pairs. Site educators reported that the time required to organize sessions meant that they often had little or no time for planning and practising delivery of the modules. Observation data and some staff interviews suggested that this lack of practice time was particularly challenging when staff had limited experience in group work.

Researcher observation of the modules and site educator feedback forms indicated that site educators generally delivered activities as described in the manual. However, less time than was recommended was spent on some of the key exercises due to staff finding them difficult to deliver and/or not well received by groups. One such example was the ‘review since the previous session’ exercise at the beginning of each module.

Also, while researcher observation and staff feedback showed fidelity of CASCADE psychological techniques was good across sessions in half the sites, it was not optimal in the remainder. Difficulties in delivering the intervention particularly occurred when sessions had groups of participants with a wide age range or group numbers were very small.

The first group that we ran had two girls and a boy and the boy was at the younger end of the teenage years and the girls were at the older, it was unfortunate because we didn't have that many patients as part of the study so it was very difficult then to get the groups sorted out so we kind of had to put them together. […] He was just a bit of a silly boy in that…I don't mean horribly, he was lovely, but just kind of played the fool a little bit whereas the girls were older and a similar age and a lot more grown up about it all. (Site educator)

Staff reported that the organization and delivery of the intervention was affected by the research context in a number of ways. First, having to restrict the education groups to a subset of recruited patients, instead of offering them to the entire clinic list, was perceived as making the organization of the groups more challenging. This meant that natural groupings of patients (by age or geographical area) often proved too difficult to achieve. Second, delays encountered in the recruitment of families to the trial in many sites (see^12^ for detail on this), meant site educators often had to wait several months after their training before they could start to organize groups and deliver the intervention. Third, some site educators reported that additional trial-related tasks, such as organizing research blood samples added to their workload and took time away from organization of, and preparation for, groups.

### Uptake and acceptability of the modules

Of the 180 young people recruited to the intervention arm, only 55 (30%) received the full education program of four modules with just over half of the original recruits (53%) attending at least one module. Eighty-four young people (47%) failed to attend any modules. Those who attended had significantly lower mean baseline HbA1c scores than those who were offered the sessions but did not attend (9.52 vs 10.33, p<0.01). Significantly more children (8–12 years) attended at least one module compared with teenagers (13–16 years; 64% vs 44%, p<0.01). Clinics were permitted to offer sessions at a time of their choice. If out of school hours sessions were not offered, the main reason given for young people not attending modules was that they did not want to miss school. For parents, taking time off work during the day was a barrier to attendance. Other reasons for non-attendance cited by children and parents included holidays and other extracurricular activities.

On most occasions a parent/carer attended with the young person. Parents and young people reported that joint attendance was a very positive aspect of the experience (see table 3). Staff also, in most instances, found it helpful to include parents.

**Table 3:** Acceptability of CASCADE to parents and young people attending at least one CASCADE module (12-month questionnaire)

Themes	Young people	Parents/carers
	‘Quite a lot’ or ‘A great deal’ n/N (%)	‘Quite a lot’ or ‘A great deal’ n/N (%)
Group dynamic		
Liked parents/young people being together in modules	81/90 (90)	81/84 (96)
Felt learnt something from other people in the group	64/93 (69)	60/85 (71)
Teaching style and length		
Liked the way the trainers taught	74/93 (81)	81/86 (94)
Felt the sessions were too long	22/94 (23)	7/85 (8)
Content		
Felt that some of the things covered were too complicated	7/92 (8)	8/85 (9)
Felt that some of the things covered they knew before	48/91 (53)	42/86 (49)

### Perceptions of impact

The majority of parents and young people who attended CASCADE groups described some positive impacts, including improved family relationships, wider knowledge and understanding of diabetes, greater confidence, and increased motivation to manage the disease (see table 4 and young person's comment below).

I've been more happier…yeah, like around the house I've been more happier. Not so many strops…'cause my readings are better and we've been given a lot more information about the ketones and how to treat it….I found it really good. [Young person]

**Table 4:** Parents’ and young people's perceptions of influence of CASCADE (12-month questionnaire)

Questionnaire items	Answered ‘Quite a lot’/‘A great deal’	
*Question: After attending some or all of the CASCADE diabetes education sessions, how much did your child/you…?*	Parent N=90	Young person N=97
Knowledge		
Understand better how insulin works	66 (74%)	70 (73%)
Understand better which foods contain CHO	73 (81%)	68 (70%)
See why counting the CHO in the food your child/you eat(s) can be helpful	80 (90%)	73 (75%)
Intention to change		
Want to stop your child's/your glucose levels from going too low or high	85 (94%)	85 (87.5%)
Want to test your child's/your BG levels more often	56 (43%)	40 (42%)
Control		
Feel more in charge of your child's/your diabetes	61 (69%)	65 (68%)
Feel able to change your child's/your insulin dose when they are exercising	68 (77%)	66 (69%)
Feel you are able to control your child's/your BG levels better	70 (78%)	64 (67%)
Access to care		
Feel more able to ring/contact your diabetes nurse/GP/hospital if your child/ you need(s) help	72 (82%)	52 (54%)
Family dynamic		
Feel you had a better understanding of how diabetes affects your family	67 (75%)	69 (72%)

BG, blood glucose; CASCADE, Child and Adolescent Structured Competencies Approach to Diabetes Education; CHO, carbohydrate; GP, general practitioner.

A number of young people and parents mentioned that timing of the CASCADE sessions would be more appropriate and useful sooner after diagnosis; site educators also commented that this may lead to better uptake of the sessions and have greater impact.

I felt they were of little use to me as I already knew everything however this kind of session would be useful to someone who had just been diagnosed. (Young person)

They're a bit sort of more ‘do as they're told’ for the first 12 months, they're more likely to attend and perhaps take it on board, it gets them in the right frame of mind early. (Site educator)

Twenty-four months after the intervention, when asked in the questionnaire what effect the program had had, nearly half of the young people selected the response “The sessions made me want to try harder and I have carried on trying”*.* However, these impacts were not reflected in the primary or secondary outcome measures, even for the subgroup of those who attended.

## Discussion

The CASCADE intervention aimed to train PDSNs and other members of diabetes teams to deliver a manualised, structured education program, based on behavior change methods, to groups of families. Training of these site educators took place over 2 days. Few members of the MDT, other than PDSNs, attended the training. Trainee educators expressed enthusiasm for the program but highlighted concerns including that: CASCADE would increase their workload; there would be practical constraints to setting up and running groups; and that incorporating the CASCADE psychological model into their practice would be challenging.

Following delivery of CASCADE in the sites, PDSNs and other clinical staff were positive about the program. Having PDSNs and dietitians, who knew the patients, as site educators worked well for both the educators and families. There were, however, feasibility issues with regard to running the program in its current form in the ‘real world’ of the National Health Service. These were evidenced by low uptake by families and staff feeling unacceptably burdened by organizational aspects of the intervention. Organizing groups was, as anticipated by staff, challenging and time-consuming and many groups did not comprise the recommended number or age range of young people. This affected group dynamics and made it difficult to run the sessions as set out in the manual. It was also difficult to keep a group together for the planned four modules. Delivery of the modules was further compromised by: the gap in time between training and delivering sessions; time spent on organizing group sessions at the expense of practising delivery of the modules; and finding some exercises consistently hard to deliver.

Despite the fact that families and staff reported that they liked the program and felt that it offered benefits, the trial found no evidence of impact on venous HbA1c at 12 and 24 months and little evidence of benefits on secondary outcomes, even with the subgroup who attended the training. We think the reasons behind this are twofold. First the organizational difficulties that made the intended group composition problematic and second the difficulties with delivery, especially the lack of fidelity to the psychological techniques. To address these issues, and to support the development of other structured education programs, we make a range of recommendations.

### Recommendations

To reduce the burden on the site educators more members of the MDT, including consultants, could attend the program training to foster greater buy-in and a team approach to facilitate sharing of the workload. To make this feasible, including containing cost, training of teams could be conducted at local sites rather than centrally in London. Furthermore, dedicated administrative support to organize venues, appointments, groups, and effective reminder systems would increase the likelihood of improved overall uptake, and would help with grouping the young people by age, as intended. Additional support for site educators in practising and sustaining quality of delivery would have been beneficial. Possible approaches could include: those associated with the successful DAFNE program,^14^ such as longer training, a greater focus in the training on improving group work skills, and an observation of CASCADE experts delivering the program; site level mentoring from CASCADE experts including feedback on site educators delivering trial runs; face-to-face mentoring from local colleagues, such as psychologists. In addition, before undertaking structured education programs, there may be a need to improve the knowledge base of some of the current pediatric diabetes service workforce, as levels of knowledge were very variable. Raising knowledge levels may be addressed by the development of a curriculum for professionals specifically in diabetes, ranging from a core curriculum (basic knowledge that all team members would be expected to know) to an extended curriculum (covering high level application of knowledge specific to individual team members). This finding may have relevance to other medical specialisms where structured education programs are being considered.

The uptake of the education sessions was low. For families the key issue was the challenge of fitting attendance into busy day-to-day routines. The education modules were offered in sessions independent of routine clinic appointments. Our data suggest that to improve accessibility it could have been advantageous to make the modules an integral part of routine clinic appointments, thereby overcoming the need for families to make additional hospital visits, with the implications this has for time away from school and work. This would require those in organizational administrative roles to assist with sustainable organizational adjustments required for extending clinic services. This finding and the suggestion that there should be greater ‘buy-in’ from the wider clinic team echo those in the broader literature on group-based programs.^15^ Furthermore in the study, participants had to have been diagnosed with diabetes for more than a year to meet the inclusion criteria for participation. Our data suggest that if the program was offered to families sooner after the initial diabetes diagnosis, this might lead to improved motivation to attend the groups. Additionally offering this structured group education more universally might be more successful, including making the organization of groups by age more feasible, than targeting those with the poorest control of their blood glucose levels. It may be more realistic to assume that those with the very poorest control might also require the greater flexibility and intensity that individualized interventions with a psychologist would offer. A summary of the key recommendations is presented in box 1.

Box 1 **Summary of key recommendations to improve training in, and delivery of, structured education sessions**

- More involvement of the wider clinical team facilitated by local training;
- Greater mentoring of site educators by trainers;
- Practice sessions with feedback from trainers for site educators before going ‘live’ and time between training and delivery of first session kept to a minimum;
- More diabetes-specific training for the pediatric diabetes service workforce to guarantee a basic level of diabetes knowledge prior to training in the program;
- Dedicated administrative support to assist with organizing the sessions;
- Education sessions to be held within clinic time;
- Offer the sessions to all young people on clinic lists and soon after diagnosis.

### Strengths and limitations of the study

It is a strength of the study that the process evaluation was unusually extensive and fully integrated into the main trial. Data were collected from all key stakeholders through a range of different methods throughout the different phases of the implementation of the intervention. Triangulation of findings enabled an evaluation of the implementation, barriers, and facilitators in relation to all aspects of implementation, operation, and perceived impact to be examined. It was also a strength that as a pragmatic RCT this intervention was evaluated in ‘real-life’ and representative settings. One limitation of the study was the impact of the research context on implementation, but steps were taken in the information and reassurance provided, methods, and timing of data collection to minimize effects as much as possible. Additionally, a major hindrance to the intervention was the lower than expected number of CASCADE groups run and the poor uptake of these groups by families. This might suggest a weakness in the intervention's pilot, which was not carried out within the same clinical contexts as the main trial. As such, opportunities to address challenges in organization and delivery were missed prior to, or through carefully managed processes within, the full trial.^16^ Experience from pragmatic studies of complex interventions such as CASCADE has yielded valuable new learning on the importance of particular investment in the developmental and piloting stages of complex interventions.^17^

## Conclusion

The extensive multimethod process evaluation showed that the CASCADE structured education program was deliverable; however, improvements in clinical and administrative support, staff training, program content, and service structures to improve accessibility for families were required. The suggested improvements identified in this study all have resource implications, and thus any future research requires cost-benefit considerations. These findings give valuable information on what is required not only in CASCADE but also other similar programs to achieve their aims.
